# Comparative transcriptomics provides novel insights into the mechanisms of selenium accumulation and transportation in tea cultivars (*Camellia sinensis* (L.) O. Kuntze)

**DOI:** 10.3389/fpls.2023.1268537

**Published:** 2023-10-02

**Authors:** Qinghua Zheng, Lina Guo, Jianyan Huang, Xinyuan Hao, Xiaoman Li, Nana Li, Yueqi Wang, Kexin Zhang, Xinchao Wang, Lu Wang, Jianming Zeng

**Affiliations:** Key Laboratory of Biology, Genetics and Breeding of Special Economic Animals and Plants, Ministry of Agriculture and Rural Affairs, National Center for Tea Plant Improvement, Tea Research Institute, Chinese Academy of Agricultural Sciences, Hangzhou, China

**Keywords:** tea cultivars, high selenium-enrichment ability, transcriptome analyses, Na_2_SeO_3_, Na_2_SeO_4_

## Abstract

Tea plants (*Camellia sinensis*) show discrepancies in selenium accumulation and transportation, the molecular mechanisms of which are not well understood. Hence, we aimed to conduct a systematic investigation of selenium accumulation and transportation mechanisms in different tea cultivars via transcriptome analysis. The Na_2_SeO_3_ and Na_2_SeO_4_ treatments improved selenium contents in the roots and leaves of three tea cultivars. The high selenium-enrichment ability (HSe) tea cultivars accumulated higher selenium contents in the leaves than did the low selenium-enrichment ability (LSe) tea cultivars. Transcriptome analysis revealed that differentially expressed genes (DEGs) under the Na_2_SeO_3_ and Na_2_SeO_4_ treatments were enriched in flavonoid biosynthesis in leaves. DEGs under the Na_2_SeO_3_ treatment were enriched in glutathione metabolism in the HSe tea cultivar roots compared to those of the LSe tea cultivar. More transporters and transcription factors involved in improving selenium accumulation and transportation were identified in the HSe tea cultivars under the Na_2_SeO_3_ treatment than in the Na_2_SeO_4_ treatment. In the HSe tea cultivar roots, the expression of sulfate transporter 1;2 (*SULTR1;2*) and *SULTR3;4* increased in response to Na_2_SeO_4_ exposure. In contrast, ATP-binding cassette transporter genes (*ABC*s), glutathione *S*-transferase genes (*GST*s), phosphate transporter 1;3 (*PHT1;3*), nitrate transporter 1 (*NRT1*), and 34 transcription factors were upregulated in the presence of Na_2_SeO_3_. In the HSe tea cultivar leaves, ATP-binding cassette subfamily B member 11 (*ABCB11*) and 14 transcription factors were upregulated under the Na_2_SeO_3_ treatment. Among them, *WRKY75* was explored as a potential transcription factor that regulated the accumulation of Na_2_SeO_3_ in the roots of HSe tea cultivars. This study preliminary clarified the mechanism of selenium accumulation and transportation in tea cultivars, and the findings have important theoretical significance for the breeding and cultivation of selenium-enriched tea cultivars.

## 1 Introduction

Selenium (Se) is an essential trace element in the human body that plays an important role in immune regulation and disease prevention (Xiang et al., 2022). Organic selenium has higher bioavailability and fewer toxic side effects in humans than those caused by inorganic selenium such as Na_2_SeO_4_ and is an important means for Se uptake (Wang et al., 2022b). Plants are important organic selenium sources for the human body; they uptake inorganic selenium from the soil through their roots and convert it into absorbable organic selenium (Ren et al., 2022). People mainly consume Se-rich grains and horticultural crops as a source of Se (Ye et al., 2015; Chao et al., 2022). Nevertheless, the global distribution of Se resources in the soil is extremely uneven. More than 15% of the world population suffers from Kashin–Beck disease and Keshan disease, and approximately 72% of the soil in China is Se-deficient (Chen et al., 2022). Therefore, in low-Se areas, Se biofortification techniques such as soil fertilization and foliar spraying are utilized to enhance Se uptake in plants (Zhang et al., 2013; Xiong et al., 2019; Zhao et al., 2019; Huang et al., 2020). Meanwhile, the Se contents of plants were also increased by cultivating the Se-enrichment cultivars (Cabannes et al., 2011).

The main forms of Se in the soil are selenate and selenite, and their ratio in the soil is controlled mainly by soil redox state and pH (Elrashidi et al., 1987; Wang et al., 2022a). The accumulation and transportation of these two forms of Se differ among plants; then, both are eventually metabolized into Se compounds (Raina et al., 2021). In higher plants, the uptake of Na_2_SeO_4_ occurs mainly through sulfate transport into the plant, which is assimilated by the sulfur assimilation pathway (El Mehdawi et al., 2018; Yu et al., 2019). Studies on the model organism *Arabidopsis thaliana* have found that sulfate transporters (SULTR1;1 and SULTR1;2) participate in Na_2_SeO_4_ transport, and SULTR1;1 increases resistance to Na_2_SeO_4_ (Yoshimoto et al., 2002; Barberon et al., 2008). On the contrary, the accumulation and transportation mechanisms of Na_2_SeO_3_ in plants are relatively complex and currently unclear. A previous study found that Na_2_SeO_3_ entered plants primarily via passive diffusion (Shrift and Ulrich, 1969). However, Broyer et al. found that an increase in phosphate concentration can inhibit plant root accumulation of Na_2_SeO_3_ (Hopper and Parker, 1999). The accumulation of Na_2_SeO_3_ is an active process in plant roots, with a similar accumulation mechanism to that of phosphorus, and both phosphate transporters (OsPHT1;2 and OsPHT1;8) are involved in Na_2_SeO_3_ accumulation and transportation in rice (Li et al., 2008; Zhang et al., 2014). There is also evidence that the aquaporin NIP2;1 aids in the accumulation of Na_2_SeO_3_ in rice (Zhao et al., 2010). Recent studies have shown that nitrate transporter (NRT1.1B) promotes the transport of selenomethionine (SeMet) in rice (Zhang et al., 2019). However, the molecular mechanisms of Se accumulation and transportation in various tea cultivars with high and low Se-enrichment abilities have not been vastly investigated. As tea can be an important Se source, it is necessary to select and breed tea cultivars with high Se-enrichment ability and explore the discrepant mechanisms between high and low Se accumulation.

As an important cash crop, the tea plant (*Camellia sinensis* (L.) O. Kuntze) has a high ability to enrich Se (Ren et al., 2022). Research on Se in tea plants has mainly focused on the effects of exogenous Se on the quality and yield of tea, the effects of soil factors on the accumulation of Se, and the mechanism of Se tolerance (Tang et al., 2012; Zhou et al., 2015). Expression analysis of genes related to Se accumulation and transportation in tea has mainly focused on sulfate and phosphate transporters. CsSULTR1;1, CsSULTR1;2, and CsSULTR1;3 have been suggested to play important roles in Se accumulation (Zhang et al., 2022). Cao et al. found that CsPHTs might play vital roles in Na_2_SeO_3_ accumulation, transportation, and homeostasis (Cao et al., 2018; Cao et al., 2021). During transcriptome analysis, *PHT3;1a*, *PHT1;3b*, *PHT1;8*, and *NIP2;1* were found to be upregulated under the Na_2_SeO_3_ treatment (Ren et al., 2022). However, there are few reports on the regulatory network and function of Se accumulation and transportation in tea plants. Hence, in this study, we aimed to gain more insights into the roles of key genes by systematically analyzing the accumulation and transportation mechanisms of Se in various tea cultivars.

In this study, three tea cultivars with contrasting Se accumulation capacities were used as experimental materials to explore by transcriptome analysis the key genes related to Se accumulation and transportation. The research provided a theoretical basis for the selection and breeding of high Se-enrichment ability tea cultivars.

## 2 Materials and methods

### 2.1 Plant material and treatments

Various cultivars were grown in Hangzhou and Enshi and bred continuously for 3 years from 2019 to 2022 as part of a pioneer field experiment. The field experiment results showed that the varieties ‘Zhongcha xicha 1 hao (XC 1)’ and ‘Zhongcha xicha 4 hao (XC 4)’ had high selenium-enrichment ability (HSe) and that the variety ‘CT2009-0025’ had low selenium-enrichment ability (LSe). Therefore, three cultivars were selected as the experimental materials for this study. One-year-old tea plant cuttings were cultured in 1/4 nutrient solution (macronutrients mmol/L: N 2.0, P 0.07, K 0.6, Mg 0.67, Ca 0.53, and Al 0.07; microelements µmol/L: Fe 4.2, Mn 1, Zn 0.67, Cu 0.13, B 7, and Mo 0.33). The plants were grown in a greenhouse with 12-h illumination, at 25°C and 70% relative humidity. The nutrient solution was replaced once weekly until new roots emerged. The cuttings were then transferred to treatment solutions without Se or supplemented with 5 µmol/L of Na_2_SeO_3_ or Na_2_SeO_4_ for 15 days. Each treatment comprised four biological replicates. Mature leaves and roots were removed for transcriptome analyses, immediately frozen in liquid nitrogen, and stored at −80°C.

### 2.2 Detection of Se contents in roots and leaves

Roots were washed with Milli-Q water containing 2 mmol/L of MES and 1 mmol/L of CaSO_4_, and the surface water was dried with absorbent paper. The roots and leaves were freeze-dried for 48 h in a lyophilizer (TF-FD-1; Zhejiang Nade Scientific Instrument Co., Ltd., Hangzhou, China). The total Se contents were determined as follows: 0.2-g samples (accurate to 0.0001 g) were weighed in a digestive tube, 4 mL of nitric acid and 2 mL of hydrogen peroxide were added, and the tube was sealed in a Mars 6 microwave digestion instrument (CEM Corp., Matthews, NC, USA). The digestion was conducted as follows: heated to 130°C for 10 min, kept for 5 min, brought to 200°C in 10 min, and held for 30 min. After the samples were digested and cooled to room temperature, they were diluted with ultrapure water to 50 mL and shaken. The total Se content of the sample was determined by NexIon 300 ICP-MS (PerkinElmer Inc., Waltham, MA, USA) with a radiofrequency (RF) power of 1,100 W; the flow rate of plasma gas (Ar) was 14 L/min, and the flow rate of reaction (CH_4_) was 0.90 mL/min. The Rpq value was 0.8, and the atomization gas flow rate was 0.98 L/min.

### 2.3 RNA extraction, library construction, and sequencing

RNA was extracted from the roots and leaves of tea plants using an RNAprep Pure Polysaccharide Plant Total RNA Extraction Kit (Tiangen Biotech, Beijing, China). RNA integrity was verified by RNase-free agarose gel electrophoresis, and RNA purity was quantified using a NanoDrop 2000 (Thermo Fisher Scientific, Waltham, MA, US). First, a sequencing library was generated using the NEBNext® Ultra™ RNA Library Prep Kit for Illumina® (New England Biolabs, Ipswich, MA, USA), and sequencing was performed on the HiSeq platform (Illumina, San Diego, CA, USA) to obtain transcriptome data. Second, the raw data obtained by sequencing were filtered and checked for sequencing error rates and guanine–cytosine (GC) content distribution to obtain clean reads to ensure good-quality, reliable data analysis. FASTP software was used to filter the raw sequence. The filtered data were sufficiently clean for subsequent analysis. Sequencing was performed by Novogene Technology Co., Ltd. (Beijing, China). In this study, the whole genome of Longjing 43 was selected as the reference sequence, and the reference genome and annotation files were downloaded from the National Genomics Data Center (https://ngdc.cncb.ac.cn/search/?dbId=gwh&q=GWHACFB00000000). Finally, the HISAT2 software was used to compare clean reads with the reference genome quickly and accurately to obtain location information of the reads on the reference genome (Pertea et al., 2015).

### 2.4 Transcriptome data analysis

The DESeq2 package of R software was used to analyze differentially expressed genes (DEGs) between the Na_2_SeO_3_ vs. CK and Na_2_SeO_4_ vs. CK (Love et al., 2014). A negative binomial distribution was used to calculate the hypothesis test probability (*p*-value), and the obtained *p*-value was corrected using Benjamini and Hochberg’s method of controlling the false discovery rate. In pairwise comparison, DEGs were clustered with *padj* < 0.05 and |log_2_FoldChange| > 0.5. The Novogene platform (https://magic.novogene.com/customer/main#/homeNew) was used to construct a Venn diagram. Kyoto Encyclopedia of Genes and Genomes (KEGG) enrichment analyses were performed for all DEGs as previously described (Wang et al., 2019b).

### 2.5 Validation of RNA-Seq results by qRT-PCR

To verify the accuracy of the transcriptome data, 10 DEGs in the roots and leaves were selected for qRT-PCR, and gene-specific primers were designed using Oligo7. The primer sequences are listed in **Supplementary Table 1**. RNA was extracted from tea roots and leaves using the RNAprep Pure Polysaccharide Plant Total RNA Extraction Kit (Tiangen), followed by the PrimeScript RT Reagent Kit (Takara Bio, Kosatsu, Shiga, Japan) to reverse transcription of 1 μg of RNA into cDNA. A 10-fold dilution of cDNA was used for qRT-PCR of the target genes using a SYBR Green I Master kit (Roche, Basel, Switzerland). *CsPTB* was used as the reference gene (Hao et al., 2014).

### 2.6 Statistical analysis

All data were analyzed by SPSS Statistics v. 26 (IBM Corp., Armonk, NY, USA), and one-way ANOVA followed the least significant difference (LSD) test at *p* ≤ 0.05. Column plots were constructed using the GraphPad Prism 8 software (GraphPad Software, La Jolla, CA, USA).

## 3 Results

### 3.1 Effects of Na_2_SeO_3_ and Na_2_SeO_4_ on Se contents in tea cultivars

**Figure 1:**
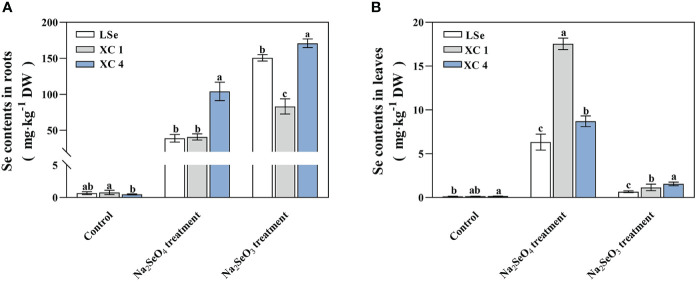
Total selenium contents in the roots and leaves of tea plants treated with Na_2_SeO_3_ and Na_2_SeO_4_. **(A)** Selenium contents in roots. **(B)** Selenium contents in leaves. Different lowercase letters above the bar indicate significant differences at the *p* < 0.05 level in the same treatment.

Total Se was measured in the roots and leaves of various HSe and LSe tea varieties treated with 5 µmol/L of Na_2_SeO_3_ or Na_2_SeO_4_ for 15 days (**Figure 1**). The total Se contents of tea plants in the roots and leaves were significantly increased by either the Na_2_SeO_3_ or Na_2_SeO_4_ treatment compared to those of the control plant treated without Se. Compared with the Na_2_SeO_4_ treatment, the Na_2_SeO_3_ treatment significantly increased the total Se in the roots and significantly decreased the total Se in the leaves. When treated with Na_2_SeO_3_ or Na_2_SeO_4_, the total Se in the roots of ‘XC 4’ was significantly higher than that in the roots of ‘LSe’, and the total Se in the leaves of either ‘XC 4’ or ‘XC 1’ was significantly higher than that in the leaves of ‘LSe’. Among them, the total Se in the leaves of ‘XC 1’ was the highest when treated with Na_2_SeO_4_, reaching 17.53 mg/kg. When treated with Na_2_SeO_3_, the total Se in the leaves of ‘XC 4’ was the highest, reaching 1.56 mg/kg.

### 3.2 Identification and KEGG enrichment analysis of DEGs in response to Na_2_SeO_3_ and Na_2_SeO_4_

**Figure 2:**
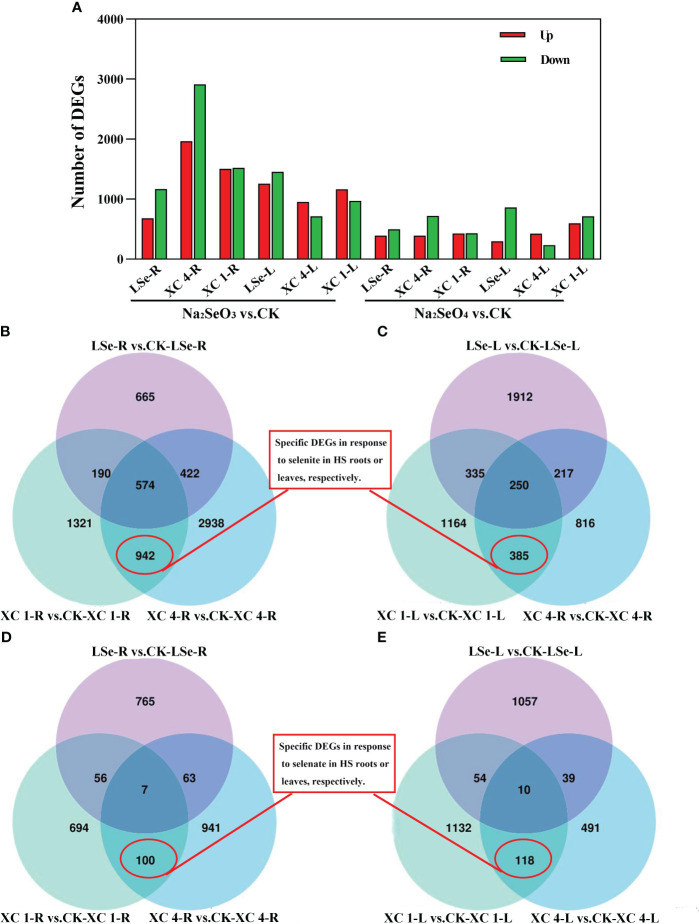
The numbers of DEGs and Venn diagram analyses between Na_2_SeO_3_ vs. control and Na_2_SeO_4_ vs. control in the three cultivars. **(A)** The numbers of upregulated and downregulated DEGs. **(B, C)** Venn diagram showing DEGs of Na_2_SeO_3_ treatment in the three cultivars roots and leaves. **(D, E)** Venn diagram showing DEGs of Na_2_SeO_4_ treatment in the three cultivars roots and leaves. ‘XC 1-R’ and ‘XC 4-R’ represent the root tissues of ‘Zhongcha xicha 1 hao’ and ‘Zhongcha xicha 4 hao’, respectively. ‘XC 1-L’ and ‘XC 4-L’ represent the leaf tissues of ‘Zhongcha xicha 1 hao’ and ‘Zhongcha xicha 4 hao’, respectively. ‘LSe-R’ and ‘LSe-L’ represent the root and leaf tissues of ‘CT2009-0025’, respectively. CK represents no selenium treatment. DEGs, differentially expressed genes.

To elucidate the discrepancies in the molecular mechanisms of Se accumulation and transportation between the HSe and LSe tea cultivars, relative genes in untreated hydroponic ‘LSe’, ‘XC 4’, and ‘XC 1’ were compared to those subjected to 5 μmol/L of Na_2_SeO_3_ or Na_2_SeO_4_ for 15 days; the criteria for screening were |log_2_FoldChange| > 0.5 and *padj* < 0.05 (**Figure 2A**). The number of genes responding to the Na_2_SeO_3_ treatment in the roots and leaves of HSe and LSe tea cultivars was higher than that responding to the Na_2_SeO_4_ treatment. When the plants were treated with Na_2_SeO_3_, there were 683, 1,964, and 1,506 upregulated genes in the roots and 1,258, 954, and 1,164 upregulated genes in the leaves in ‘LSe’, ‘XC 4’, and ‘XC 1’ cultivars, respectively. The number of downregulated genes in the roots was 1,168, 2,912, and 1,521 and in the leaves was 1,456, 714, and 970 in ‘LSe’, ‘XC 4’, and ‘XC 1’, respectively. When the plants were treated with Na_2_SeO_4_, the number of upregulated genes in ‘LSe’, ‘XC 4’, and ‘XC 1’ roots was 392, 391, and 427, whereas that in the leaves was 298, 424, and 599, respectively; the number of downregulated genes in the roots was 499, 720, and 430 and in the leaves was 862, 234, and 715 in ‘LSe’, ‘XC 4’, and ‘XC 1’, respectively. Notably, the number of DEGs after treatment with Na_2_SeO_4_ was lower than that after the Na_2_SeO_3_ treatment in all three cultivars, indicating higher sensitivities of tea plants to Na_2_SeO_3_ than to Na_2_SeO_4_.

A Venn diagram was used to analyze the DEGs of HSe and LSe tea cultivars under Se treatment. Under the Na_2_SeO_3_ treatment, the number of DEGs in the roots and leaves of the HSe tea cultivars was 942 and 385, respectively (**Figures 2B, C**), indicating that these genes responded to Na_2_SeO_3_ in the HSe tea cultivars. Under the Na_2_SeO_4_ treatment, the number of DEGs in the roots and leaves of the HSe tea cultivars was 100 and 118, respectively (**Figures 2D, E**), indicating that these genes responded to Na_2_SeO_4_ in the HSe tea cultivars. By comparing different tissues under the same treatment, the related tissue-specific DEGs responding to Se were determined. Analysis of the UpSet map showed that 748 and 288 genes were tissue-specific (**Supplementary Figure 1A**) and responded separately to Na_2_SeO_3_ in the roots and leaves of the HSe tea cultivars, respectively. Similarly, 83 and 105 genes (**Supplementary Figure 1B**) responded separately to Na_2_SeO_4_ in the roots and leaves of HSe tea cultivars, respectively, indicating tissue specificity.

**Figure 3:**
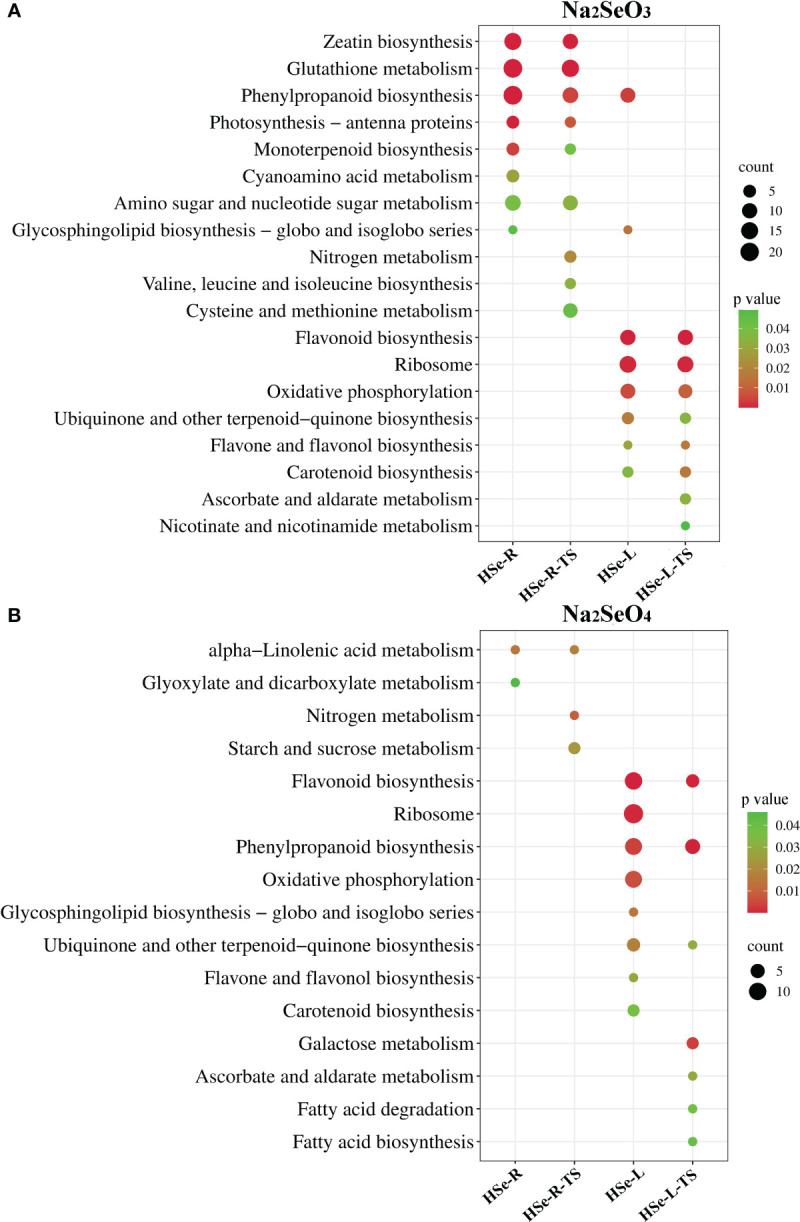
KEGG pathway analysis of DEGs in response to Na_2_SeO_3_ and Na_2_SeO_4_ treatment. **(A)** Treatment with Na_2_SeO_3_. **(B)** Treatment with Na_2_SeO_4_; HSe-R and HSe-L represent KEGG enrichment analyses of DEGs in the roots and leaves of HSe tea cultivars, respectively. HSe-R-TS and HSe-L-TS represent KEGG enrichment of tissue-specific DEGs of the HSe tea cultivars in roots and leaves, respectively. KEGG, Kyoto Encyclopedia of Genes and Genomes; DEGs, differentially expressed genes.

KEGG enrichment analysis was performed on the DEGs in response to Se and the tissue-specific DEGs in the roots and leaves of HSe tea cultivars. Under the Na_2_SeO_3_ treatment, KEGG enrichment analysis was performed on 942 DEGs and 748 tissue-specific DEGs in the roots of the HSe tea cultivars. The DEGs were significantly enriched with the glutathione metabolism, zeatin biosynthesis, and phenylpropanoid biosynthesis pathways (**Figure 3A**). KEGG enrichment analysis was performed on 385 DEGs and 288 tissue-specific DEGs in the leaves of HSe tea cultivars, and the DEGs were significantly enriched with the flavonoid biosynthesis and ribosome pathways (**Figure 3A**). Similarly, KEGG enrichment analysis was performed of 100 DEGs and 83 tissue-specific DEGs, which were associated with Na_2_SeO_4_ in the roots of HSe tea cultivars, and it was found that they were significantly enriched with α-linolenic acid metabolism p (**Figure 3B**). The 118 DEGs and 105 tissue-specific DEGs in the leaves of the HSe tea cultivars were mainly enriched with flavonoid biosynthesis pathways (**Figure 3B**). Notably, the enriched pathways of DEGs in the roots of HSe tea cultivars in response to the Na_2_SeO_4_ and Na_2_SeO_3_ treatments were different, whereas the DEGs in the leaves of HSe tea cultivars were mainly enriched with the flavonoid biosynthesis pathways under both treatments.

### 3.3 DEGs involved in Se accumulation and transportation of the HSe tea cultivars

**Figure 4:**
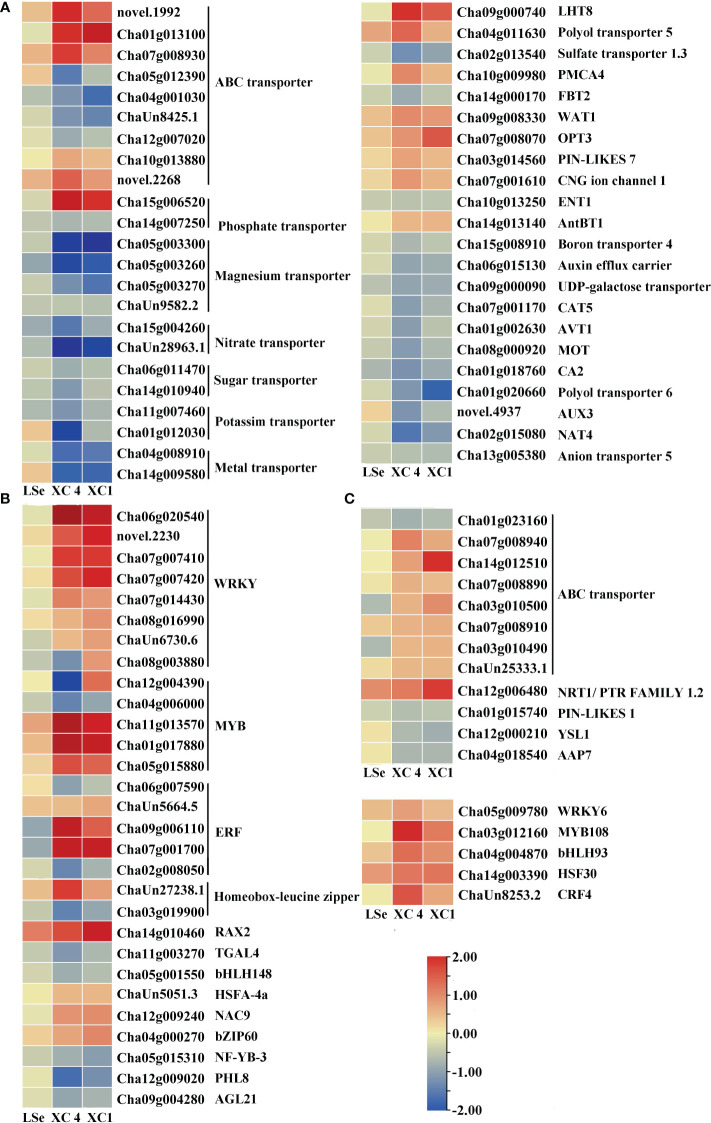
Heatmap of putative Se transporters and transcription factors identified as DEGs that responded to Na_2_SeO_3_ in the roots of the HSe tea cultivars. **(A, B)** The heatmap of tissue-specific DEGs related to transporters and transcription factors in the roots of HSe tea cultivars. **(C, D)** The heatmap of DEGs related to transporters and transcription factors in the roots of HSe tea cultivars. DEGs, differentially expressed genes; HSe, high selenium-enrichment ability.

**Figure 5:**
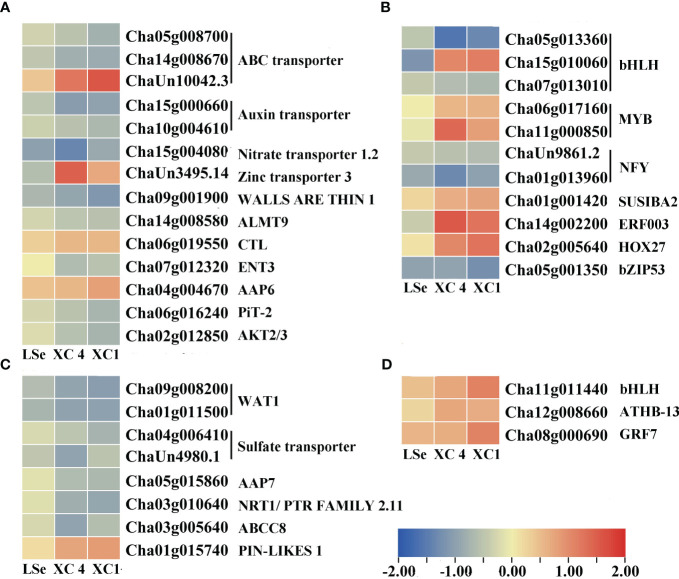
Heatmap of putative Se transporters and transcription factors identified as DEGs that responded to Na_2_SeO_3_ in the leaves of the HSe tea cultivars. **(A, B)** The heatmap of tissue-specific DEGs related to transporters and transcription factors in the leaves of HSe tea cultivars. **(C, D)** The heatmap of DEGs related to transporters and transcription factors in the leaves of HSe tea cultivars. DEGs, differentially expressed genes; HSe, high selenium-enrichment ability.

The transcriptome analysis identified 33 and 63 transporters in the leaves and roots, respectively (**Supplementary Tables 2**, **3**). Among them, 22 and 11 transporters in the leaves, and 57 and six transporters in the roots responded to the Na_2_SeO_3_ and Na_2_SeO_4_ treatments, respectively. Under the Na_2_SeO_4_ treatment, *ChaUn24016.1* (*PHT1;4*) in the leaves and *Cha09g005000* (*SULTR1;2*) and *Cha03g006400* (*SULTR3;4*) in the roots were identified in HSe tea cultivars. The roots of the HSe tea cultivars had more DEGs in response to the Na_2_SeO_3_ treatment than in response to the Na_2_SeO_4_ treatment. The transporters responding to Na_2_SeO_3_ mainly included ABC transporters, magnesium transporters, sugar transporters, phosphate transporters, oligopeptide transporters, and lysine histidine transporters (**Figures 4**, **5**), which may be beneficial for exploring the key genes involved in Na_2_SeO_3_ accumulation and transportation in HSe tea cultivars. Seventeen ABC transporters were identified in the roots of HSe tea cultivars, of which nine were tissue-specific. There were 12 upregulated genes and five downregulated genes, of which *Cha01g013100* (*ABCG11*) was upregulated by 2.01- and 2.85-fold in ‘XC 4’ and ‘XC 1’, respectively. Among the two phosphate transporters, *PHT3;1* was upregulated by 2.92- and 1.99-fold in ‘XC 4’ and ‘XC 1’, respectively. *Cha12g006480* (*NRT1*) was upregulated by 1.18- and 1.85-fold in ‘XC 4’ and ‘XC 1’, respectively.

Meanwhile, the upregulated genes related to transferase, cytochrome P450, dehydrogenase, reductase, and ABC transporter genes, which were significantly differentially expressed in the HSe tea cultivars compared with their expression in the LSe tea cultivar, were filtered twofold in roots in response to Na_2_SeO_3_ (**Table 1**). Notably, nine glutathione transferases were involved in response to Na_2_SeO_3_, of which the expression of *ChaUn6696.4* (*GSTU17*) was 4.40- and 2.73-fold higher in ‘XC 4’ and ‘XC 1’, respectively. Moreover, nine genes were related to glucosyltransferases, of which *ChaUn5691.2* (crocetin-glucosyltransferase) expression was 4.49- and 2.69-fold higher in ‘XC 4’ and ‘XC 1’, respectively, than in LSe. Additionally, *Cha01g009120* (*ABCG8*) and *Cha13g008780* (*ABCC4*) were also identified. We also found that in two enzymes in the ethylene synthesis pathway, *Cha06g014280* (1-aminocyclopropane-1-carboxylate oxidase 1) expression was 2.83- and 3.06-fold higher in ‘XC 4’ and ‘XC 1’, respectively, than in LSe, and *Cha12g001220* (1-aminocyclopropane-1-carboxylate synthase) expression was 2.08- and 1.13-fold higher in ‘XC 4’ and ‘XC 1’, respectively, than in LSe. However, the co-upregulated genes in response to Na_2_SeO_3_ in the leaves were mainly heat shock proteins, auxin-responsive protein IAA7, and glycine-rich protein 2 (**Table 2**). In particular, heat shock proteins were obviously upregulated more in the HSe cultivars than in LSe by 2.18- and 1.94-fold for ‘XC 4’ and ‘XC 1’, respectively.

**Table 1:** The co-upregulated DEGs in the roots of HSe and LSe cultivars under Na_2_SeO_3_ treatment.

Gene ID	Description	LSe log_2_FC (+Se^4+^ vs -Se^4+^)	XC 4 log_2_FC (+Se^4+^ vs -Se^4+^)	XC 1 log_2_FC (+Se4+ vs -Se4+)	XC 4/LSe log_2_FC/log_2_FC	XC 1/LSe log_2_FC/log_2_FC
Transferase						
Cha05g011690	Probable glutathione *S*-transferase	0.84	1.89	1.73	2.25	2.06
ChaUn6696.4	Glutathione *S*-transferase U17	1.99	8.74	5.43	4.40	2.73
Cha08g013620	Probable glutathione *S*-transferase	0.73	2.13	1.76	2.92	2.41
Cha08g013640	Probable glutathione *S*-transferase	0.87	2.51	2.04	2.90	2.35
Cha01g021070	Glutathione *S*-transferase U10	1.74	4.28	3.46	2.46	1.99
ChaUn15528.2	Glutathione *S*-transferase U8	1.07	2.60	2.95	2.43	2.75
Cha08g013610	Probable glutathione *S*-transferase	1.23	2.85	2.60	2.31	2.11
Cha05g011780	Probable glutathione *S*-transferase	0.95	2.17	1.91	2.28	2.00
Cha03g014180	Microsomal glutathione *S*-transferase 3	0.53	1.35	0.89	2.55	1.69
ChaUn5691.2	Crocetin glucosyltransferase, chloroplastic	0.54	2.43	1.46	4.49	2.69
ChaUn5691.1	Crocetin glucosyltransferase, chloroplastic	1.24	5.31	3.90	4.28	3.15
ChaUn9614.2	Beta-d-glucosyl crocetin beta-1,6-glucosyltransferase	1.33	2.93	2.48	2.21	1.87
Cha01g002120	Beta-d-glucosyl crocetin beta-1,6-glucosyltransferase	0.93	2.36	2.44	2.52	2.62
Cha13g010050	Raucaffricine-*O*-beta-d-glucosidase	0.55	1.25	0.75	2.26	1.36
Cha01g022200	Phenolic glucoside malonyltransferase 2	0.75	1.61	1.14	2.16	1.53
ChaUn16290.1	UDP-glycosyltransferase 73C6	0.72	2.41	2.17	3.33	3.00
Cha06g010160	UDP-glycosyltransferase 71K1	1.38	3.74	3.01	2.71	2.18
Cha06g013540	UDP-glycosyltransferase 73C3	0.65	1.54	0.99	2.35	1.51
ChaUn17166.1	BAHD acyltransferase	1.21	3.18	2.27	2.63	1.88
ChaUn12774.5	Probable lipid transfer	1.46	3.19	2.30	2.18	1.57
Cha08g010320	Tyrosine aminotransferase	1.84	3.93	3.99	2.14	2.17
Cha02g015780	Scopoletin glucosyltransferase	1.46	3.08	2.59	2.11	1.78
Cytochrome P450						
Cha14g003030	Cytochrome P450 CYP72A219	1.21	3.18	1.89	2.63	1.56
ChaUn5945.1	Cytochrome P450 94A2	0.74	2.79	2.96	3.75	3.98
ChaUn13615.4	Cytochrome P450 CYP72A219	0.74	3.10	0.97	4.22	1.31
Cha04g001400	Cytochrome P450 83B1	0.92	1.95	1.33	2.12	1.44
Cha14g011380	Cytochrome P450 89A9	0.95	2.26	1.54	2.38	1.62
Dehydrogenase and reductase						
ChaUn22024.2	Short-chain type dehydrogenase/reductase	0.54	2.20	1.09	4.05	2.01
Cha13g001990	Probable mannitol dehydrogenase	2.63	6.71	5.58	2.55	2.12
Cha06g007800	(+)-Neomenthol dehydrogenase	1.09	2.77	2.54	2.53	2.32
Cha14g007840	(+)-Neomenthol dehydrogenase	1.18	2.79	2.62	2.35	2.21
Cha14g007830	Short-chain dehydrogenase/reductase 2b	2.44	6.52	5.50	2.68	2.26
ChaUn20934.2	Salutaridine reductase	2.09	4.33	3.75	2.08	1.80
ABC transporter						
Cha01g009120	ABC transporter G family member 8	1.43	2.87	2.04	2.00	1.42
Cha13g008780	ABC transporter C family member 4	1.37	2.94	1.97	2.14	1.44
ACC oxidative synthase						
Cha06g014280	1-Aminocyclopropane-1-carboxylate oxidase 1	1.05	2.96	3.19	2.83	3.06
Cha12g001220	1-Aminocyclopropane-1-carboxylate synthase	1.51	3.15	1.71	2.08	1.13
Transcription factor						
Cha11g001130	Ethylene-responsive transcription factor ERF071	1.03	2.49	3.55	2.40	3.43

DEGs, differentially expressed genes; HSe, high selenium-enrichment ability; LSe, low selenium-enrichment ability.

**Table 2:** The co-upregulated DEGs in the leaves of HSe and LSe cultivars under Na_2_SeO_3_ treatment.

Gene ID	Description	LSe log_2_FC (+Se^4+^ vs -Se^4+^)	XC 4 log_2_FC (+Se^4+^ vs -Se^4+^)	XC 1 log_2_FC (+Se^4+^ vs -Se^4+^)	XC 4/LSe log_2_FC/log_2_FC	XC 1/LSe log_2_FC/log_2_FC
Cha03g013630	15.7 kDa heat shock protein	0.62	1.35	1.20	2.18	1.94
ChaUn9222.6	Thaumatin-like protein 1	0.74	1.37	1.25	1.84	1.68
Cha12g002960	Auxin-responsive protein IAA7	0.74	1.26	0.91	1.72	1.24
Cha06g001000	Glycine-rich protein 2	0.56	0.95	0.77	1.70	1.37
novel.5594	Wound-induced basic protein	0.58	0.93	1.12	1.61	1.94
ChaUn7167.6	E3 ubiquitin protein ligase RIE1	0.51	1.04	0.80	2.02	1.57
Cha06g006700	Protein transport protein Sec61 subunit beta	0.75	1.35	0.81	1.79	1.07
Cha13g008460	Phospholipase D zeta 2	0.57	0.99	0.84	1.75	1.48
Cha01g017260	Alpha/beta hydrolase family	0.64	1.07	0.94	1.68	1.47

DEGs, differentially expressed genes; HSe, high selenium-enrichment ability; LSe, low selenium-enrichment ability.

### 3.4 DEGs involved in Se regulatory network of the HSe tea cultivars

Five and four transcription factors responded to Na_2_SeO_4_ in the roots and leaves of the HSe tea cultivars, respectively (**Supplementary Table 4**). However, 34 and 14 transcription factors were significantly expressed in the roots and leaves of the HSe tea cultivars, respectively, in response to Na_2_SeO_3_ (**Supplementary Table 5**). Among them, *Cha07g001700* (ethylene-responsive transcription factor (ERF) 1B) was upregulated by 2.98- and 2.85-fold in the roots of ‘XC 4’ and ‘XC 1’, respectively; *Cha09g006110* (*ERF110*) was upregulated by 3.36- and 1.49-fold in the roots of ‘XC 4’ and ‘XC 1’, respectively; *Cha14g002200* (*ERF003*) was significantly upregulated in leaves. With the Na_2_SeO_3_ treatment, the *Cha11g013570* (*MYB44*), *Cha01g017880* (*MYB75*), and *Cha05g015880* (*MYB80*) transcription factors in roots were significantly upregulated, while the *Cha11g00085* (*MYB12*) and *Cha06g017160* (*MYB1R1*) transcription factors in the leaves were significantly upregulated. In addition, many *WRKY* transcription factors were identified in response to the Na_2_SeO_3_ treatment. For example, *Cha07g007420* (*WRKY6*), *Cha07g007410* (*WRKY42*), *novel.2230* (*WRKY51*), and *Cha06g020540* (*WRKY75*) were significantly upregulated in the roots of HSe tea cultivars. In particular, *WRKY75* was upregulated by 3.42- and 5.10-fold in ‘XC 1’ and ‘XC 4’, respectively. Only one *WRKY* transcription factor responded to Na_2_SeO_3_ in the leaves.

We also identified upregulated genes in three tea cultivars. Among them, *Cha11g001130* (*ERF071*) showed a higher induction degree in response to Na_2_SeO_3_ in the ‘XC 4’ and ‘XC 1’ than that in LSe by 2.40- and 3.43-fold, respectively.

### 3.5 DEGs involved in glutathione metabolism of the HSe tea cultivars

**Figure 6:**
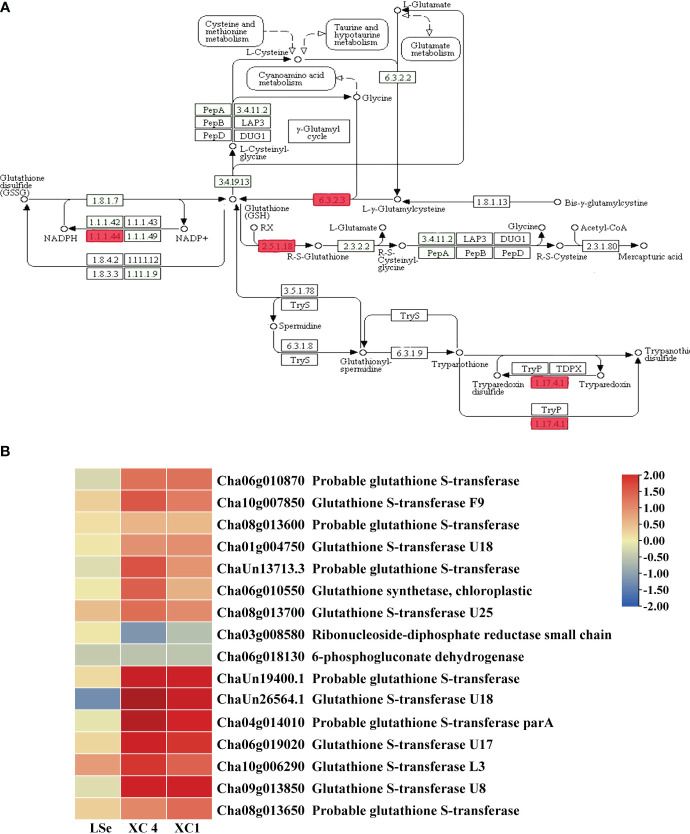
The DEGs in the roots of tissue-specific HSe tea cultivars are involved in the glutathione metabolic pathway. **(A)** Glutathione metabolic pathway. **(B)** Heatmap of differential genes. Red rectangles represent DEGs. ‘XC 4’ and ‘XC 1’ represent tissue-specific DEGs identified during analysis of Na_2_SeO_3_-treated vs. control samples in the roots of ‘XC 4’ and ‘XC 1’, respectively. Different colors indicate different levels of gene expression based on log_2_FoldChange. DEGs, differentially expressed genes; HSe, high selenium-enrichment ability.

KEGG enrichment analysis of root tissue-specific DEGs after the Na_2_SeO_4_ treatment showed enrichment primarily of the glutathione metabolic pathway. Therefore, we analyzed DEG patterns related to glutathione metabolism (**Figure 6A**). Sixteen root tissue-specific DEGs were identified in the glutathione metabolic pathway of the HSe tea cultivars (**Figure 6B**). There were 13 DEGs in the glutathione *R*-transferase (2.5.1.18) pathway, and all of them were upregulated. Among them, *Cha09g013850* (glutathione *S*-transferase (*GST*) *U8*), *ChaUn26564.1* (*GSTU18*), and *Cha04g014010* (glutathione *S*-transferase part A) were upregulated more than twofold in the HSe tea cultivars (**Supplementary Table 6**). Hence, we speculated that *GST* genes enhanced the accumulation of Se in the roots of Se-enrichment tea plants and participated in the detoxification of Se.

### 3.6 Validation of DEGs by qRT-PCR

**Figure 7:**
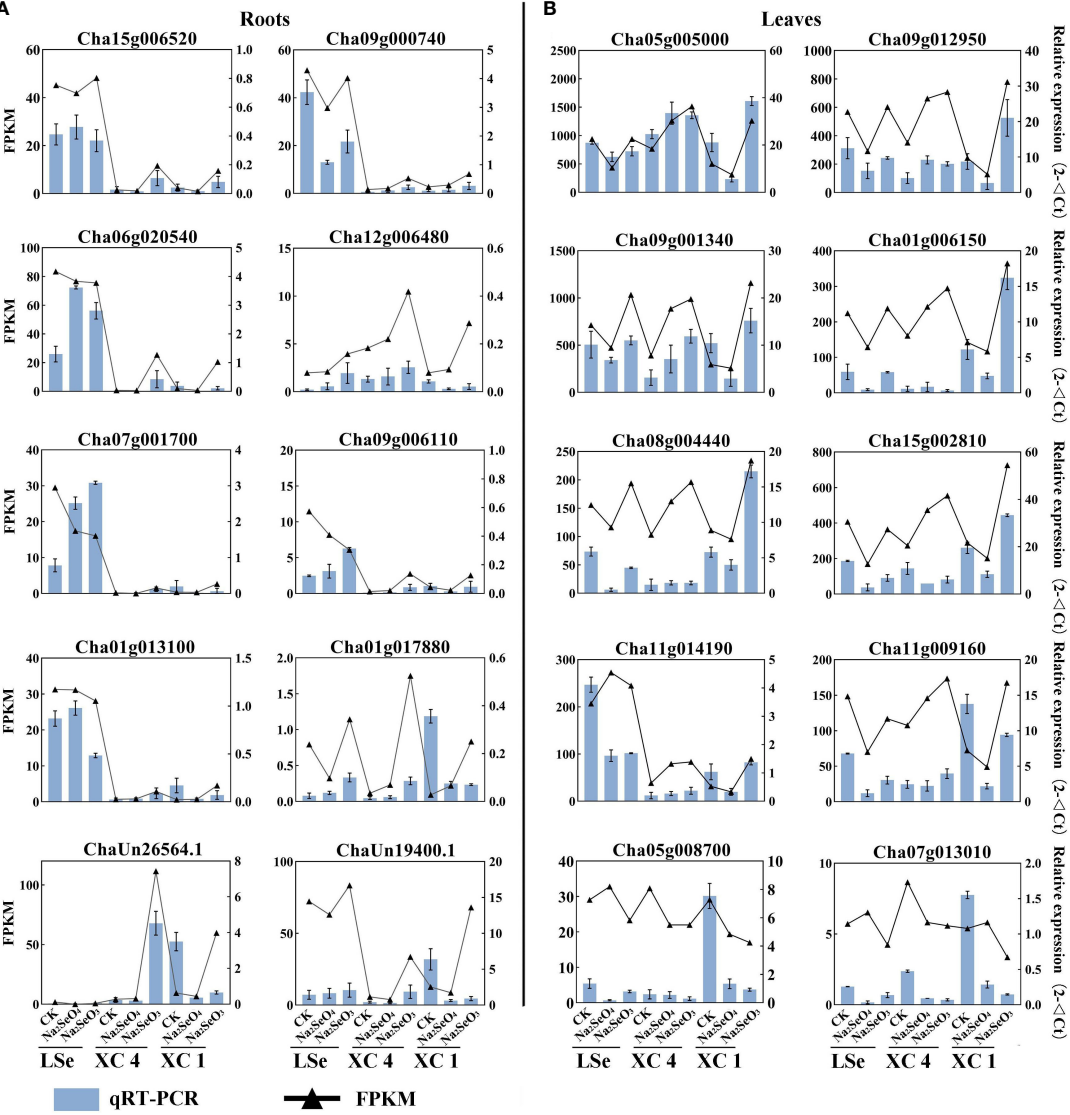
The expression of DEGs in roots **(A)** and leaves **(B)**. The black line represents expression levels as detected by RNA-Seq. The blue bars represent expression as detected by qRT-PCR. The error bar represents the standard deviation. DEGs, differentially expressed genes.

To verify the accuracy and reliability of the RNA-Seq data, we screened 10 genes related to transporters and transcription factors for qRT-PCR analysis in root and leaf tissues. The expression patterns demonstrated by RNA-Seq and qRT-PCR were consistent for all the tested 10 genes in the roots and leaves, and the results are shown in **Figure 7**. Therefore, the RNA-Seq data can be considered reliable.

## 4 Discussion

Based on their Se accumulation ability, plants can be divided into non-accumulator, Se-indicator, and Se-accumulator species (White, 2016). Tea plants have strong Se enrichment abilities, and various tea cultivars show significant differences in their ability to accumulate Se. Tea plants are more likely to accumulate Na_2_SeO_3_ than Na_2_SeO_4_ in root tissues and rarely transport it to leaf tissues. Based on previous reports, the Se contents in tea leaves reached Se-enriched levels after being treated with 5 μmol/L of Se concentrations for more than 14 days (Fang and Shen, 1992; Liu et al., 2021). In our study, under the Na_2_SeO_4_ or Na_2_SeO_3_ treatment for 15 days, ‘XC 1’ had the highest Se transportation ability from roots to leaves. The roots of ‘XC 4’ were readily able to accumulate Se but translocated only small amounts to the leaves. To explore the networks that regulate Se accumulation, transcriptome analyses were performed on various Se-enriched cultivars.

### 4.1 Transporters involved in Se accumulation and transportation in HSe tea cultivars

The RNA-Seq method has been used to analyze DEGs and has become a standard in multiple research fields (Costa-Silva et al., 2023). RNA-Seq technology can be used to identify genes related to Se transportation, accumulation, and assimilation (Hu et al., 2022). The transport mechanism of Na_2_SeO_3_ is more complex than that of Na_2_SeO_4_. Na_2_SeO_3_ has different ionization forms under various pH conditions. At pH 5, 5 µmol/L of Na_2_SeO_3_ mainly exists in the form of HSeO_3_^−^ (97.2%), SeO_3_^2−^ (2.4%), and H_2_SeO_3_ (0.4%) (Zhang et al., 2010). Plants accumulate HSeO_3_^−^ via phosphate transporters and H_2_SeO_3_ via silicon transporters (Zhao et al., 2010; Wang et al., 2019a). The accumulation of SeO_3_^2−^ is partly through ion channels, and most of the mechanisms are unclear. In the study, transcriptome analysis was performed to elucidate the accumulation and transportation mechanisms of Na_2_SeO_3_ and Na_2_SeO_4_ in various Se-enriched tea cultivars.

ABC transporters share a conserved ATPase domain and catalyze ATP to supply the energy required for the transmembrane transport of substrates, thus participating in important physiological processes, such as plant secondary metabolite accumulation and biotic and abiotic stress response (Byrne et al., 2010). These transporters can be divided into eight subfamilies, from ABCA to ABCG, and ABCI (Verrier et al., 2008). In rice, ABC transporters were detected under the Na_2_SeO_3_ treatment, indicating that they might participate in Se accumulation and transportation (Kong et al., 2021). Meanwhile, the ABCB, ABCC, and ABCG transporters have been suggested to be involved in the accumulation and transport of Se in cowpeas (Li et al., 2023). Fourteen upregulated *ABC* genes were also found in our study (**Figure 4**), including *ABCA*, *ABCB*, *ABCC*, and *ABCG*, indicating that they may play vital roles in the accumulation and transportation of Se in tea roots.

Se mainly exists in organic forms, including selenoproteins, Se-polysaccharides, and Se-nucleotides (Zhou et al., 2020). However, there are few reports on the accumulation and transportation of Se-polysaccharides. In this study, sugar metabolites related to the abundance of glucosyltransferase genes in HSe cultivar roots were upregulated in response to Na_2_SeO_3_ (**Table 1**). Hence, we speculate that glucosyltransferase genes may participate in the formation and accumulation of Se-polysaccharides in tea plants.

In plants, sulfate transporters are involved in the accumulation and transportation of Na_2_SeO_4_, whereas phosphate transporters are involved in the accumulation of Na_2_SeO_3_ (Lazard et al., 2010; Tombuloglu et al., 2017). Cao et al. found that the response number and expression level of *CsPHT* gene in tea roots increased with increasing Na_2_SeO_3_ concentration (Cao et al., 2021). We found that only one SULTR1;2 and PHT3;1 might play important roles in Na_2_SeO_4_ and Na_2_SeO_3_ uptake, respectively, by HSe cultivars. Most Na_2_SeO_3_ accumulates in the root tissues after being converted into organic selenium, making its transport to the leaf tissues difficult (Ren et al., 2022). This finding is similar to that regarding the mode of accumulation of Na_2_SeO_3_ in rice. The NRT1.1B transporter in rice enhances the ability to transfer SeMet from roots to shoots (Zhang et al., 2019). In tea plants, we found that NRT1 may play an important role in the transportation and allocation of Se.

### 4.2 Transcription factors involved in Se accumulation and transportation in HSe tea cultivars

Reactive oxygen species (ROS) production in plants promotes an increase in the jasmonic acid and ethylene stress hormone levels (Overmyer et al., 2003). In *Arabidopsis*, jasmonic and ethylene respond to Na_2_SeO_3_, which induces *ERF* to participate indirectly in the regulation of plant defense (Lorenzo and Solano, 2005). Thirty-four and 14 Na_2_SeO_3_-regulated transcription factors were identified in the roots and leaves of the HSe tea cultivars, respectively. Among these, *ERF118* and *ERF110* were significantly upregulated in the roots of HSe cultivars, and *ERF003* was significantly upregulated in the leaves. Cao et al. found that jasmonic and ethylene could regulate a defensive network by upregulating the expression levels of transcriptional factors, including *ERF* and *MYB* in tea plant roots under the Na_2_SeO_3_ treatment (Cao et al., 2018). Our study suggested that similar regulatory mechanisms of *ERF*s and *MYB*s may exist in tea plants. *WRKY*s are involved in plant nutrition stress response. For example, *WRKY75* was significantly upregulated (Devaiah et al., 2007) and positively regulated phosphate and Se accumulation under P-deficient conditions. In this study, *WRKY75* was also found to be involved in regulating the accumulation of Na_2_SeO_3_ in tea plant roots.

### 4.3 Glutathione metabolism genes involved in Se accumulation and transportation in HSe tea cultivars

Accumulation of excessive Se can lead to oxidative responses in plants, resulting in excessive ROS that induce apoptosis. Glutathione can remove the excessive ROS produced by plants to protect tissues from oxidative damage and improve plant tolerance to Se (Zou et al., 2021). After treatment with high Na_2_SeO_3_ concentrations, 15 genes involved in glutathione metabolism were identified in tea plants. Genes encoding glutathione *S*-transferase, glutathione synthetase, glutathione peroxidase, and glutathione reductase were significantly upregulated, suggesting that they may increase the tolerance and accumulation of Se in tea plants (Cao et al., 2018). In the study, 13 *GST* genes were upregulated in the roots of HSe tea cultivars treated with Na_2_SeO_3_. Furthermore, the nine co-upregulated *GST*s showed significantly higher expression in HSe cultivar roots, as compared to the LSe cultivar. GSTs might be induced to transfer SeO_4_^2−^ ions to GSH to form glutathione *S*-conjugates (GS-X) under the Na_2_SeO_3_ treatment, which can explain the accumulation of abundant Se in roots (Zhou et al., 2018). Therefore, we speculate that *GST*s are likely key genes for accumulating Se in tea plant roots.

## 5 Conclusion

**Figure 8:**
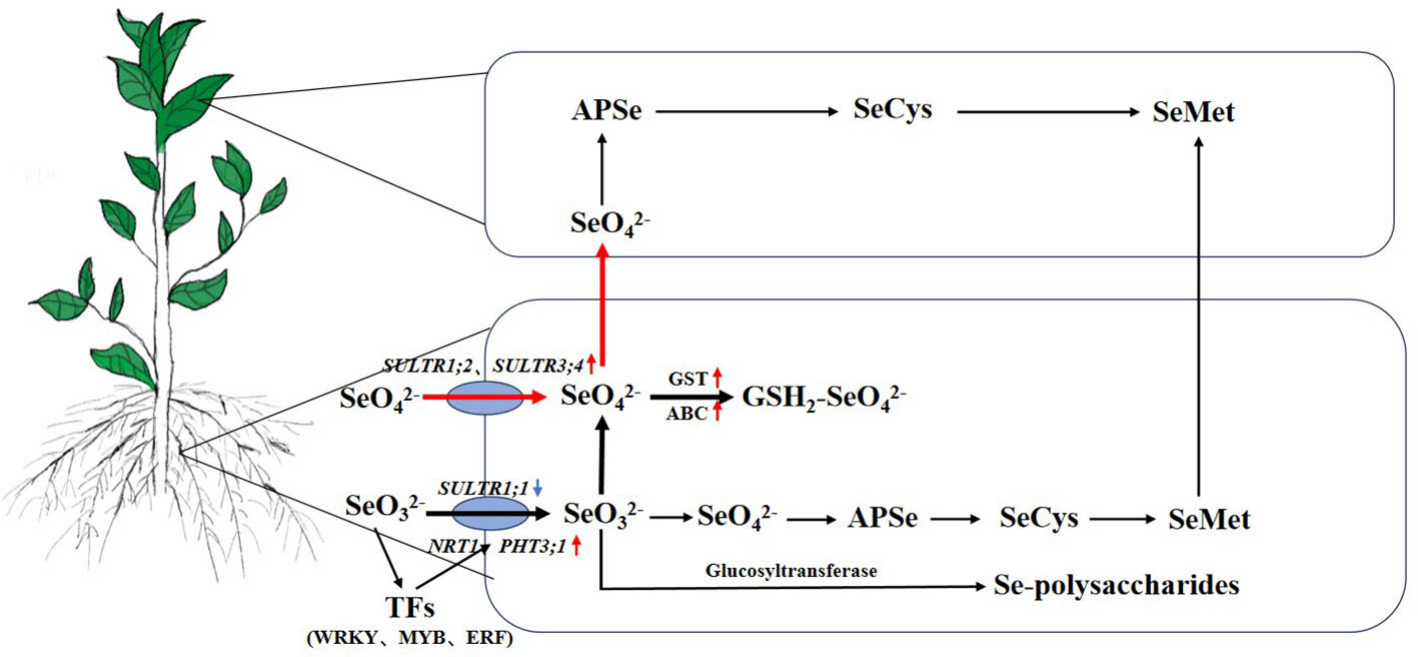
The uptake, transport, and metabolism of Se in the HSe tea cultivars. The red arrow represents upregulated genes. The blue arrow represents downregulated genes. The black bold lines represent the main metabolic pathways of Na_2_SeO_3_. The red bold lines represent the main metabolic pathways of Na_2_SeO_4_. HSe, high selenium-enrichment ability.

In this study, the effects of different Se sources on the Se contents of the roots and leaves of various high and low Se-enrichment ability tea cultivars were analyzed. Under treatment, the Se contents of roots were obviously higher than those of leaves, especially after treatment with Na_2_SeO_3_, and the Se contents of HSe cultivars in leaves were significantly higher than those in LSe leaves. The RNA-Seq analysis showed that the number of DEGs after the Na_2_SeO_3_ treatment was higher than that after the Na_2_SeO_4_ treatment, indicating that the tea plant responded more strongly to Na_2_SeO_3_. Further studies suggested that *GST*s might participate in the accumulation of abundant Se in roots. The accumulation of Na_2_SeO_3_ was increased in line with the upregulated expression levels of *ABC*, glucosyltransferases, *NRT1*, and *PHT3;1* in tea plants (**Figure 8**). Meanwhile, the expression levels of *SULTR1;2* and *SULTR3;4* genes in HSe tea cultivars were obviously induced by Na_2_SeO_4_, which participates in the accumulation of Se. These data provide a basis for the mechanism of Se accumulation and transportation in various high and low Se-enrichment ability tea cultivars.

## Data availability statement

The original contributions presented in the study are included in the article/**Supplementary Files**, further inquiries can be directed to the corresponding authors. The datasets presented in this study can be found in online repositories. The raw RNA ‒ 438 Seq generated are available in the NCBI Sequence Read Archive (SRA) database under 439 accession number SRR25823839-SRR25823909 and SRR25823916-SRR25823917.

## Author contributions

QZ: Writing – original draft. LG: Writing – original draft. JH: Writing – review & editing. XH: Software, Supervision, Writing – review & editing. XL: Methodology, Writing – review & editing. NL: Writing – review & editing. YW: Methodology, Software, Writing – review & editing. KZ: Writing – review & editing. XW: Project administration, Writing – review & editing. LW: Methodology, Supervision, Writing – review & editing. JZ: Writing – review & editing, Funding acquisition, Project administration, Supervision.
