# Supplementary figures and images for: Comparative transcriptomics provides novel insights into the mechanisms of selenium accumulation and transportation in tea cultivars (Camellia sinensis (L.) O. Kuntze)
Source: Front Plant Sci. 2023 Oct 2;14:1268537. doi: 10.3389/fpls.2023.1268537 (PMC10577196; doi:10.3389/fpls.2023.1268537)

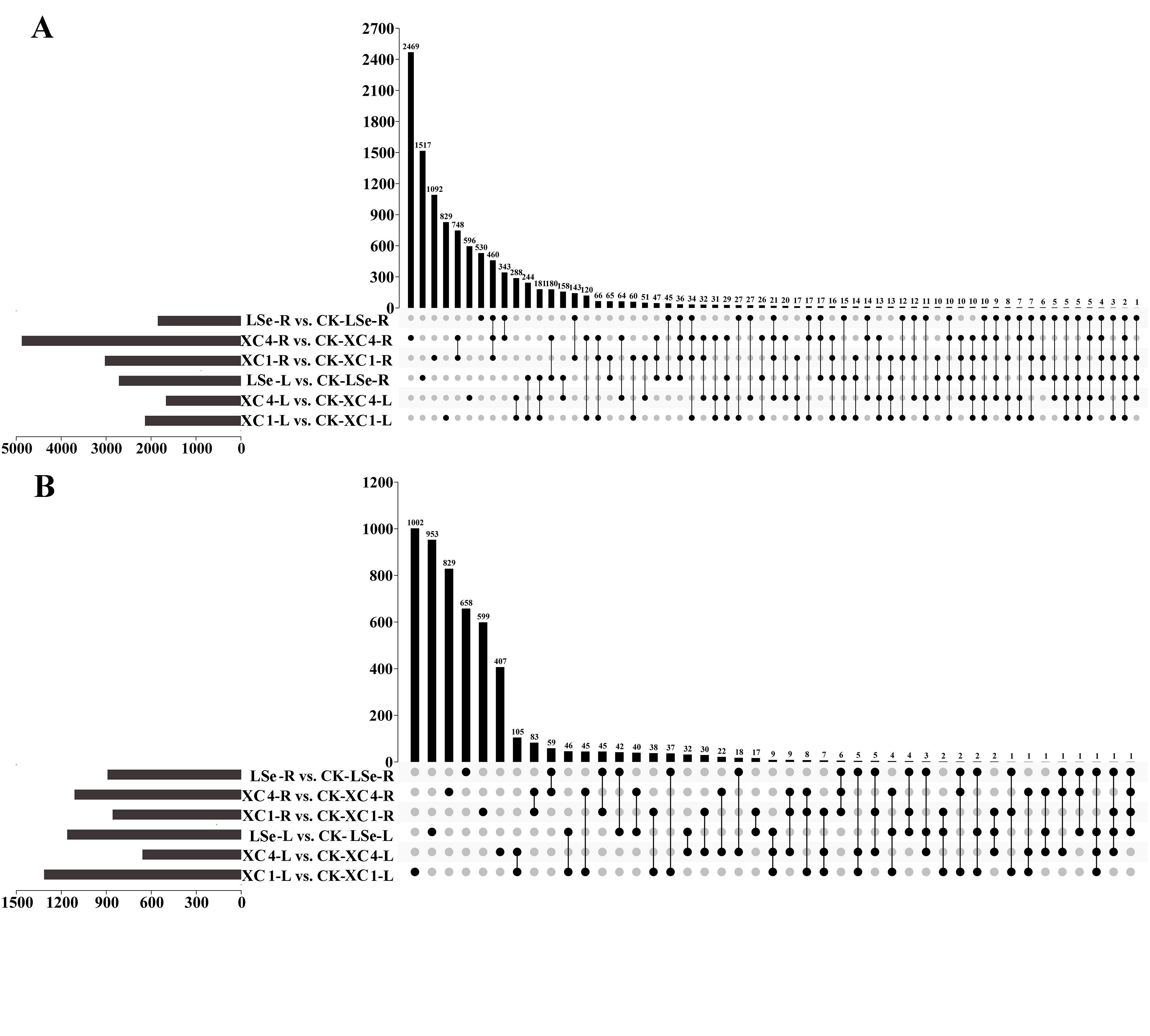

Supplement: Supplementary file 1 [file Image_1.jpg]
